# Somatic mutation profiles in aged military nuclear test veterans: A comparative whole-genome sequence study

**DOI:** 10.1371/journal.pone.0351624

**Published:** 2026-06-30

**Authors:** Justin Ofosu-Dankwa, Cristina Sisu, Rhona M. Anderson

**Affiliations:** 1 Centre for Health Effects of Radiological and Chemical Agents, Uxbridge, England; 2 Centre for Genome Engineering and Maintenance, College of Health, Medicine and Life Sciences, Brunel University London, Uxbridge, United Kingdom; Odense University Hospital, DENMARK

## Abstract

Veterans of the British nuclear testing programme represent an aged group of ex-military personnel who may have been exposed to ionising radiation through their participation at nuclear testing sites. This study aimed to compare the somatic mutational landscape of a cohort of 30 nuclear test veterans with that of an age-matched cohort of 30 control veterans. Variants were identified from publicly available whole-genome sequencing data using a bioinformatics pipeline developed in accordance with the gold standard approaches as defined by the Broad Institute. The resulting set of raw SNV and INDEL variants for each individual veteran were subjected to several filtering steps to reduce the noise arising from common mutations, before the average number and types were compared for each cohort using the Grubbs test. The genomic distribution of these variants was also examined by assessing for any mutation clustering considered characteristic of radiation exposure (SNVs and/or INDELs occurring within 10 bp) using a 10 bp running window and separately, the identification of mutational signatures by fitting SNVs to the COSMIC database Human Cancer v3.4. When comparing the nuclear test veteran and control cohorts, we found no statistically elevated frequency of any variant type or clusters. The dominant SBS signatures in both cohorts were those typically associated with ageing. A qualitative assessment of the functional impact of the most prevalently observed variants in each cohort showed these to also be associated with age. For example, in the control cohort, variants were found in LINC02098-ETS1 and RCL1, genes linked to classic age-related conditions such as hair loss and osteoarthritis. In the nuclear test veteran cohort, we observed multiple variants affecting the CHODL gene in approximately 40% of participants. CHODL encodes chondrolectin, a protein important for maintaining the structural integrity and function of tissues. In conclusion, the absence of significant genetic differences between cohorts, together with the prevalence of age-associated mutations, is consistent with ageing being one of the primary drivers of the observed somatic variation in these veterans, overshadowing any potential environmental, including historical radiation, effects.

## Introduction

Somatic mutations are present in all cells of the human body after conception and accumulate throughout life. They may arise through normal cellular functions and from exposure to a wide range of damaging agents in addition to the cellular processing events that respond to such damage. Ionising radiation, a potent genotoxin, is known to increase the mutational burden with increasing dose and although the underlying mechanisms remain unclear, increased somatic mutations and their accumulation is established as a risk factor for cancer and non-cancer diseases [1,2]. The human mutational landscape can be broadly classified into a spectrum of various types; point mutations, including single-nucleotide polymorphisms (SNPs), insertions/deletions (INDELs), structural variants, and copy number variations. Ionising radiation deposits energy in the form of structured tracks, the pattern of which determines the spatial distribution of the initial lesions and, consequently, the type and distribution of resulting mutations within the genome [3]. For example, INDELs and clustered variants—where multiple mutations occur in proximity—are considered characteristic of radiation exposure [2,4–6]. The Catalogue of Somatic Mutations in Cancer (COSMIC) is a continually expanding database of mutational signatures that not only associate specific patterns of variants with distinct cancer types but also provide insight into the DNA damage and repair processes that shape them [7,8]. Thus, characterising the mutational landscape can offer critical insight into the potential origins of somatic mutations.

British nuclear test (NT) veterans represent a population who were potentially exposed to ionising radiation in the 1950/60s [9,10]. Based on numerous studies, it is likely that the vast majority were exposed to no or low dose exposures only (low dose defined as less than 100mSv) [11]. Nonetheless there remains concerns about their own health and that of their descendants, which they believe may have been adversely affected by their involvement at these nuclear test sites [12,13]. The Genetic and Cytogenetic Family Trio (GCFT) study was the first to collect blood samples from British NT veterans and their families to investigate potential genetic or chromosomal alterations in offspring arising from historical paternal exposure to ionising radiation [10]. As part of this study, whole-genome sequencing (WGS) was performed on family trios of NT and control military veterans not present at test sites, to assess the prevalence of germline mutations [14]. No statistically significant differences were observed between the two cohorts to support an increased burden of germline mutations in the NT group [14]. However, an excess of germline single-nucleotide variants (SNVs) were assigned to mutational signature SBS16 in a small group of families (2 control and 6 NT), which correlated with cytogenetic markers of nuclear fallout exposure in veteran fathers. Although interpretation is complicated by the small sample size and the presence of control families, this raised the possibility that SBS16 could reflect molecular processing of radiation-induced damage from internalised exposure and, as such, serve as a transgenerational biomarker of paternal radiation exposure [15].

The aim of this present study was a comparative assessment of the somatic mutational landscape in aged NT and control veterans to primarily examine for any cohort differences including those which might reflect past radiation exposure of the haemopoietic compartment and/or have functional implications for veterans’ health. Specifically, we examined single-nucleotide variants (SNVs), INDELs and mutation clustering in the publicly available WGS dataset of blood sampled from NT and control cohorts. In addition, all SNVs were assigned to one of 90 recognised mutational signatures to determine whether somatic profiles in veterans resembled those previously observed in the germline.

## Materials and methods

### Ethical approval and consent to participate

Publicly available whole genome sequence data generated from the Genetic and Cytogenetic Family Trio (GCFT) study was used in this manuscript. The GCFT study was conducted in accordance with the relevant guidelines and regulations of the UK ethical framework and were approved by the UK Health Research Authority (17/LO/0273). Written informed consent was obtained from all subjects as part of the GCFT study.

### Data access

Raw sequencing data for the 60 veteran samples sequenced as part of the GCFT study was extracted from the SRA public repository (Accession: PRJNA788492). The samples were split into two groups: control and NT veterans based on the information provided in the associated metadata. The full list of the SRR codes used in data retrieval is shown in Table 1.

**Table 1:** List of NCBI accession numbers for each veteran sample in the two groups as identified in the Moorhouse *et al.* 2022 study.

Cohort	No of samples	Project: PRJNA788492 Accession Numbers
Control	30	SRR17255346, SRR17255349, SRR17255352, SRR17255356, SRR17255359, SRR17255362, SRR17255366, SRR17255369, SRR17255372, SRR17255375, SRR17255392, SRR17255395, SRR17255399, SRR17255402, SRR17255405, SRR17255408, SRR17255412, SRR17255415, SRR17255418, SRR17255422, SRR17255425, SRR17255428, SRR17255432, SRR17255435, SRR17255438, SRR17255441
NTV	30	SRR17255281, SRR17255284, SRR17255285, SRR17255288, SRR17255291, SRR17255294, SRR17255298, SRR17255313, SRR17255316, SRR17255320, SRR17255323, SRR17255326, SRR17255329, SRR17255330, SRR17255333, SRR17255336, SRR17255339, SRR17255343, SRR17255376, SRR17255379, SRR17255382, SRR17255386, SRR17255389, SRR17255409, SRR17255442, SRR17255443

All the study participants were cancer-free British residents of England or Wales, born in 1935 or later. The 30 NT were group-matched by a cohort of 30 control military veterans based on age and period of service. The median age at blood sample collection for the participants in both groups was 80 years old (Control 80.0 (76–83) and NT 79.5 (74–82)). Additional characteristics of the two cohorts are shown in Table S1 in S1 File.

### Variant calling pipeline

Variant calling was conducted using a bioinformatics pipeline developed using the tools, resources, best practices and gold standard approaches [16] defined by the Broad Institute [17] for variant discovery, as implemented in the Genome Analysis Toolkit 4 (GATK4). In summary, the raw reads were aligned to the Human Reference Genome GRCh38 using the Burrows-Wheeler aligner [18] BWA-MEM algorithm [19]. Duplicated reads were identified and marked to avoid technical artifacts and reads realigned. Next, the HaplotypeCaller algorithm [20] was used to create a first draft of raw variants (single nucleotide variants - SNVs and insertions-deletions - INDELs). The quality of each variant was evaluated using a number of thresholds as proposed by the Broad Institute Protocol [21] for variant filtering. We applied base quality score recalibration followed by variant calling to ensure confidence in the resulting variant set. The variants that passed all the filtering thresholds were collected and used in subsequent analyses. Finally, each variant was functionally annotated to indicate gene location and potential impact (e.g., amino acid change). A full list of software packages and libraries used, including their version, is provided in Table S2 in S1 File. Furthermore, the thresholds and quality control metrics used for SNPs and INDELs calling are shown in Table S3 in S1 File.

### Variant selection

**Fig 1:**
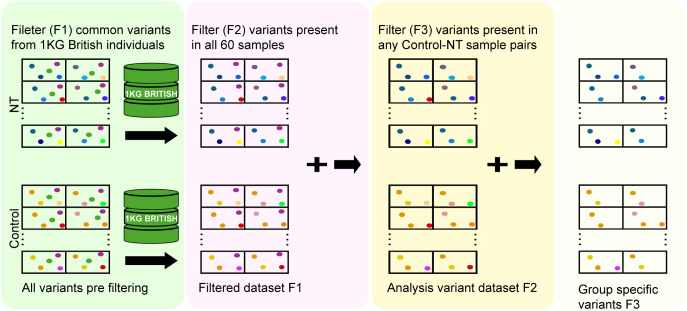
Variant selection protocol. Each block represents a veteran sample; top blocks are associated with NT and bottom blocks are representing control samples. Each coloured dot indicates a variant (INDEL or SNV). Dots of same colour illustrate the same variant present in multiple samples (e.g., forest green dots are variants present in the 1 KG dataset, purple dots are variants common to all 60 samples, etc.). At each filtering step, variant dots of the same colour are removed, and the resulting dataset is named after the applied filter (e.g., following the filtering step F1, the dataset is labelled filtered dataset F1).

The resulting set of variants for each individual veteran was subjected to several filtering steps to reduce the noise level introduced by the presence of common mutations. This was achieved using a binary pass/fail protocol: presence of a variant in the complimentary cohort indicates removal (fail) while the absence results in a pass flag. At a first attempt (filter F1), we removed common variants present in British European individuals using data from the 1000 Genomes project, at an allele frequency higher than 1% [22]. Next, the common variants which were shared between all samples in the control and NT groups were also removed to avoid signals resulting from features such as age, sex, and common life events (filter F2). The resulting set of variants was used for all further investigation (cluster analysis, mutation signatures). Lastly, a set of veteran unique variant sets were created containing only mutations found in the control or NT samples respectively (filter F3). A summary of the variant selection workflow is shown in Fig 1.

### Mutation signatures analysis

Sigflow [23] was used to identify mutational signatures from the COSMIC database Human Cancer v3.4 [24] that are present in control and NT veteran samples within the analysis variant dataset F2. Sigflow was run using the standard parameters with 1000 bootstrap runs and the hg38 reference human genome. Sigprofiler [25] was used to auto-extract signatures and allowed to identify the optimum solution (the number of COSMIC signatures that match the ones in the samples). The Tally package was used to produce a summary of the per sample and a cumulative plot overall. For each fitted signature, the statistical difference between the variant counts for the NT vs control veteran cohorts were computed using a Kruskal-Wallis test. Any result with a Bonferroni correction adjusted p-value lower than 0.05 was considered significant.

### Mutation clusters analysis

**Fig 2:**
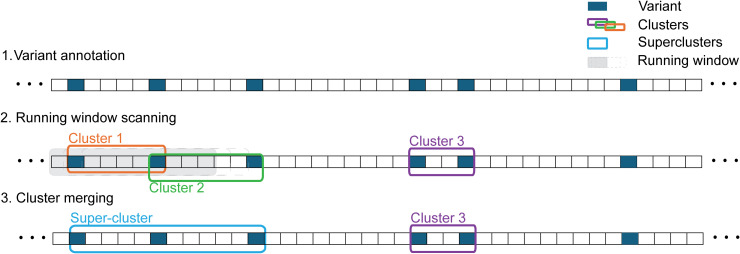
Identification of mutational clusters using a 10 base length running window on the analysis variant dataset.

The analysis variant dataset F2 was used for cluster identification. Clustered DNA (or complex clustered) damage is defined as two or more lesions (or three or more base or single-strand breaks) within 10 base-pairs [26,27]. Accordingly, mutation clusters were annotated using a 10 bp running window to scan the genome. Specifically, for each nucleotide position an interval of 10 upstream nucleotides (including the starting position) were scanned for presence of variants (both SNVs and INDELs). If two or more variants were identified, the window was labelled as “cluster”. Clusters with overlapping variants were merged into a super-cluster where any two consecutive variants are present within a 10 bp interval of each other. This expansion resulted in clusters ranging from 10 to 100 bp. No clusters larger than 100 bp were observed in these conditions for any of the analysed samples. The results from all the samples are combined to identify clustered variant rich regions across samples. A summary of the clustering process is shown in Fig 2.

### Statistical analysis

All veteran samples were scanned for statistically significant outliers with respect to the average number of incidences per cohort using the Grubbs statistical test. In absence of any suitable outlier candidates, elements larger than 1.5 SD were highlighted. All statistical tests conducted resulting in a p-value metrics were subjected to multiple hypothesis testing using Bonferroni correction. No adjustments were made for potential confounders (Table S1 in S1 File).

## Results

We explored the similarities and differences in somatic variation between two age matched cohorts of veterans that were previously sequenced as part of the GCFT study. Namely 30 aged veterans who took part in the British nuclear testing programme and a group of 30 military veterans who had served in tropical regions over the same period, but who had not been present at nuclear test sites. The two cohorts were previously described in [10].

### Identification of DNA mutations

Using the variant calling pipeline, we have identified and annotated SNVs and INDELs in each veteran sample. The resulting variant sets were subjected to further filtering as shown in Fig 1 to remove variants present in the British men part of the European Super Population of the 1000 Genomes project. No information on the specific ages of its participants was given for the 1kGP, but all participants were aged 18+ [28,29]. Furthermore, we have filtered common SNVs and INDELs shared between all the 60 veteran samples, to minimise the enrichment in variants appearing as a result of the veteran age, or common life events (F2 dataset). Finally, we defined group specific variant sets by removing variants present in any NT-control sample pair (F3 dataset). The set following filtering step F2 was deemed comprehensive enough for highly sensitive analyses such as variant cluster identification and mutational signature analysis. However, this came at the trade-off of group specificity, as in this dataset, a number of variants would be shared between samples in the control and NT cohorts, potentially increasing the background noise. To address this issue, we have employed the final F3 filter that resulted in variant sets containing only mutations specific to either the NT or control group. This dataset, exhibiting high cohort specificity, was used in functional analysis with the aim at identifying key mutated genes.

**Fig 3:**
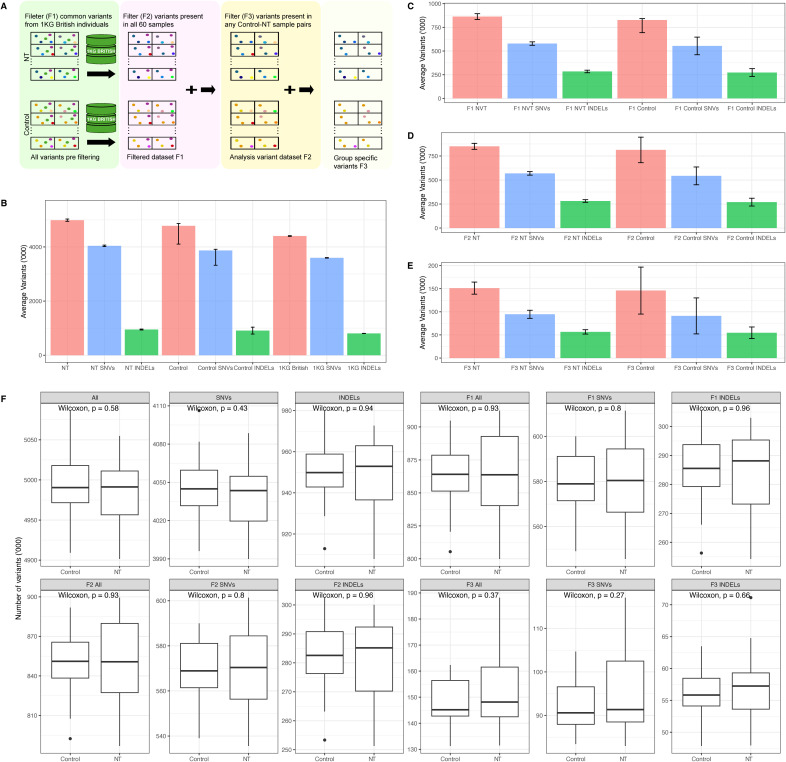
Distribution of variant count (total, SNVs and INDELs) across different filtering groups. **A.** Schematic summary of variant filtering steps (as shown in Fig 1). **B.** The average variant count across control veterans (n = 30), NT veterans (n = 30), and 1kGP British Men (n = 29) [28,29]. **C**, **D**, **E.** Average variant count for each filtering step. The y-axis represents the average value in thousands. SNVs are represented by blue bars and INDELs the green bars. Error bars depict the ± standard deviation of the counts per cohort. **F.** Boxplot of variant distribution for each filtering step between control and NT cohorts.

The outcome of variant filtering is summarised in Fig 3. On average, the number of variants in the NT veterans was slightly larger compared to the control cohort, however overall, there were no statistically significant differences between the two veteran cohorts at total or variant type level following each filtering step. A summary of the average variant count for each cohort following each filtering step is shown in Table 2.

**Table 2:** Summary of average variant numbers and types after each filtering step.

Filters	Group	Variants	SNVs	INDELs
No filter total	Test	4988404.93	4039853.44	948267.51
	Control	4778172.10	3870002.67	907897.14
F1 1 KG common variants	Test	863372.27	578955.83	284360.12
	Control	827096.01	553881.27	272947.95
F2 common variants across all 60 samples	Test	850353.60	568911.72	281386.60
	Control	813112.63	542975.64	269808.30
F3 common variants between any test-control pair	Test	151146.87	94589.48	56515.06
	Control	145885.23	91080.47	54643.91

Looking at the variant counts per sample (see Table S5 in S1 File), we observed a rather uniform distribution of total variant count, SNVs and INDELs with no statistically significant outliers detected for either the NT or control cohort. However, a number of samples (three NT and one control) were seen to be enriched with more than 1.5 SD from the mean. Namely for the F2 dataset, the samples SRR17255382, SRR17255443, SRR17255275, SRR17255305 had the largest number of SNV variants. By contrast no NT or control samples were enriched for the INDELs in the F2 dataset.

Next, we looked at variants present in annotated gene biotypes: protein coding, lncRNA and pseudogenes according to GENCODE v47 [30]. Here again we observed a relatively uniform distribution of both SNVs and INDELs across all veteran samples for the studied biotypes (see Tables S6 and S7 in S1 File). This uniformity suggests a stable presence of variants across all the annotated regions of the genome which could be related to the strong selection pressure the annotated regions are located in [31,32]. A few NT and control samples did however show a deviation larger than 1.5 SD from the mean in terms of variant count (SNV or INDEL) in one of the three biotypes annotated. Further, the number of NT veteran samples that showed SNV and/or INDEL count deviations larger than 1.5 SD from the mean in at least one biotype across the various filtered groups (all, F1, F2, F3) was consistently larger than the number of control samples exhibiting a similar pattern.

### Identification of clustered mutations

**Fig 4:**
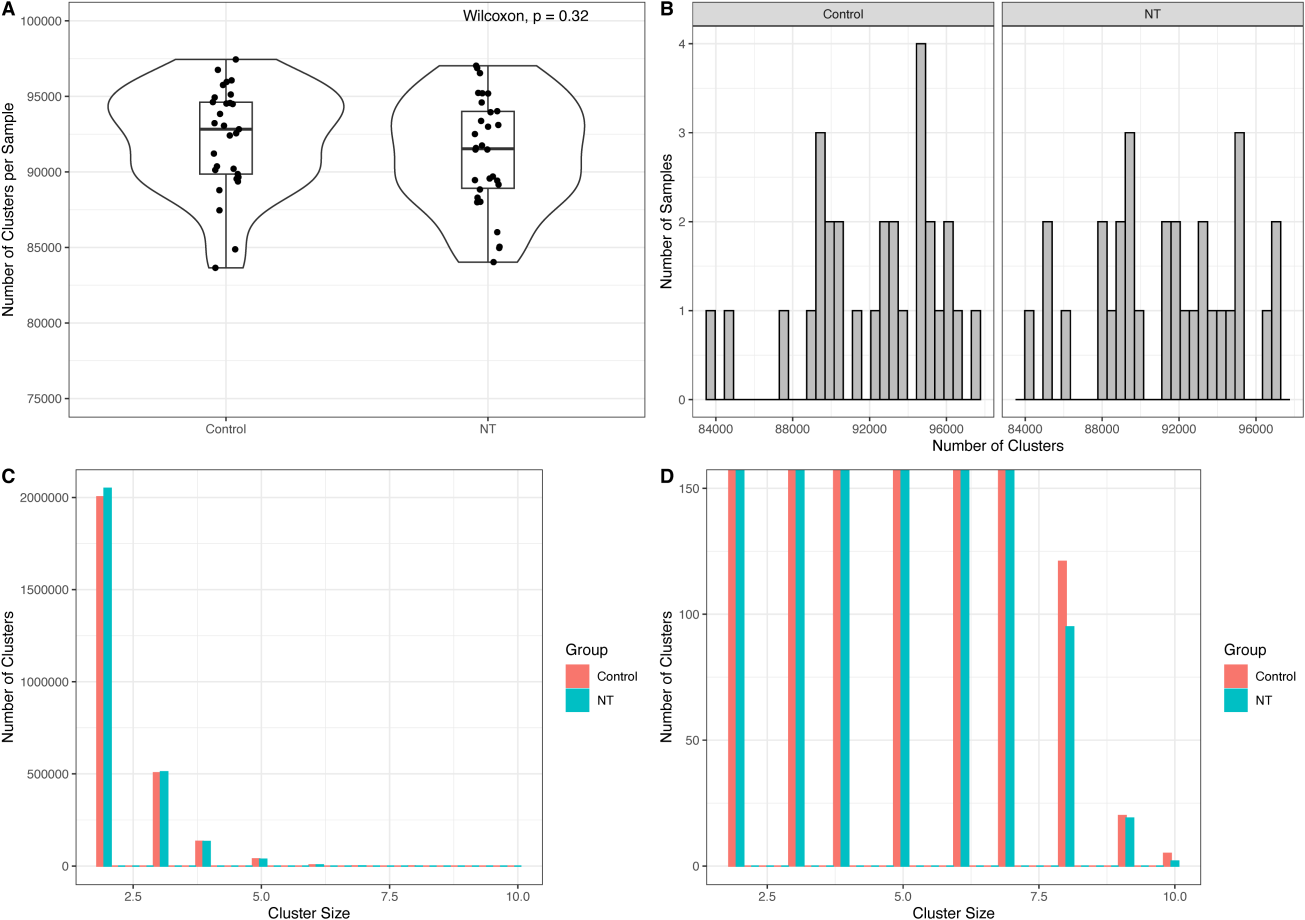
Variant cluster distribution in NT and control cohorts. **A.** Box and violin plots showing the total number of clusters per sample. **B.** In depth distribution of total clusters per sample. **C.** The distribution of clusters per number of variants. The smallest cluster contains 2 variants while the larger cluster contains 10. **D.** Zoom in distribution of the high variant count clusters between NT and control cohorts.

Clustered mutations are varyingly defined as multiple DNA lesions within 10–40 base pairs (bp) and are known to be a characteristic hallmark of exposure to ionising radiation [33,34]. Accordingly, we examined for clustered mutations in the veteran cohorts that may be indicative of exposure, using the F2 filtered dataset. The choice of this variant dataset was to allow a comprehensive image of the variant content in both cohorts specific enough to capture cohort specific signals and sensitive enough to highlight collections of mutations that contribute to various phenotypes. For this, consecutive variants separated by 10 bp or less were assigned to mutation clusters as described in Methods. A summary of the cluster’s distribution in NT and control datasets is shown in Fig 4.

Overall, we observed no significant difference in the mutational cluster profile between the two cohorts. Moreover, while the NT cohort showed a slight increase in the number of clusters containing only 2 variants, the control cohort seemed to be enriched (while not statistically significant) in large cluster sizes (with 8, 9 or 10 variants). At an individual level however, we were able to identify two NT samples (SRR17255443 and SRR17255333) that had more than 1.5 SD total clustered mutations compared to rest.

### Mutational signatures

To further elucidate the underlying mechanisms driving the observed SNV mutations, each of the SNVs identified in F2 filtered control and NT cohorts were assigned to 90 known mutational signatures from the COSMIC v3.4 database [7]. This is based on six single base substitution (SBS) types: C > A, C > G, C > T, T > A, T > C and T > G and nucleotides immediately 5’ and 3’. SNVs were fitted to all available COSMIC mutational signatures in v3.4, of these 43 mutational signatures had substantial numbers of SNVs fitted to them for all samples in both control and NT cohorts.

**Fig 5:**
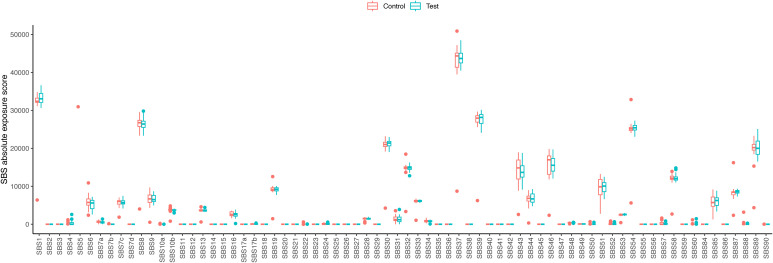
SNV mutations allocated to SBS signatures for control and NT cohorts. Data were fitted to COSMIC v3.4 database with signature fitting to one (and only one) signature. Supporting information regarding the p-values, signature instability and error values are presented in Tables S7-11 Figures S1 and S2 in S1 File.

The majority of somatic SNVs in both veteran cohorts were fitted to several visually dominant mutational signatures (SBS1, SBS5, SBS30, SBS37, SBS39, SBS54, SBS89) (Fig 5). SBS5 showed the highest counts, with over 50,000 mutations fitted to it, in both cohorts. Interestingly, SBS1 and SBS5 are known to correlate the number of mutations with age of the individuals [35], suggesting the high presence of SBS1 and SBS5 corresponds to these ageing cohorts. SBS30 is commonly described as associated with deficiency in base excision repair due to inactivating mutations in NTHL1 [36] while SBS89 is highlighted as being commonly present in samples from younger people [37]. By contrast, no information is currently available to describe the importance of the SBS37, SBS39 and SBS54 mutational signatures. Several other SBS signatures, including SBS16, had SNVs fitted to them for both cohorts, however, overall, no statistical differences were found between the two cohorts for any of the COSMIC signatures.

### Functional analysis

We explored the functional relevance, if any, of the somatic mutations identified using two different approaches.

#### Gene markers of radiation.

**Fig 6:**
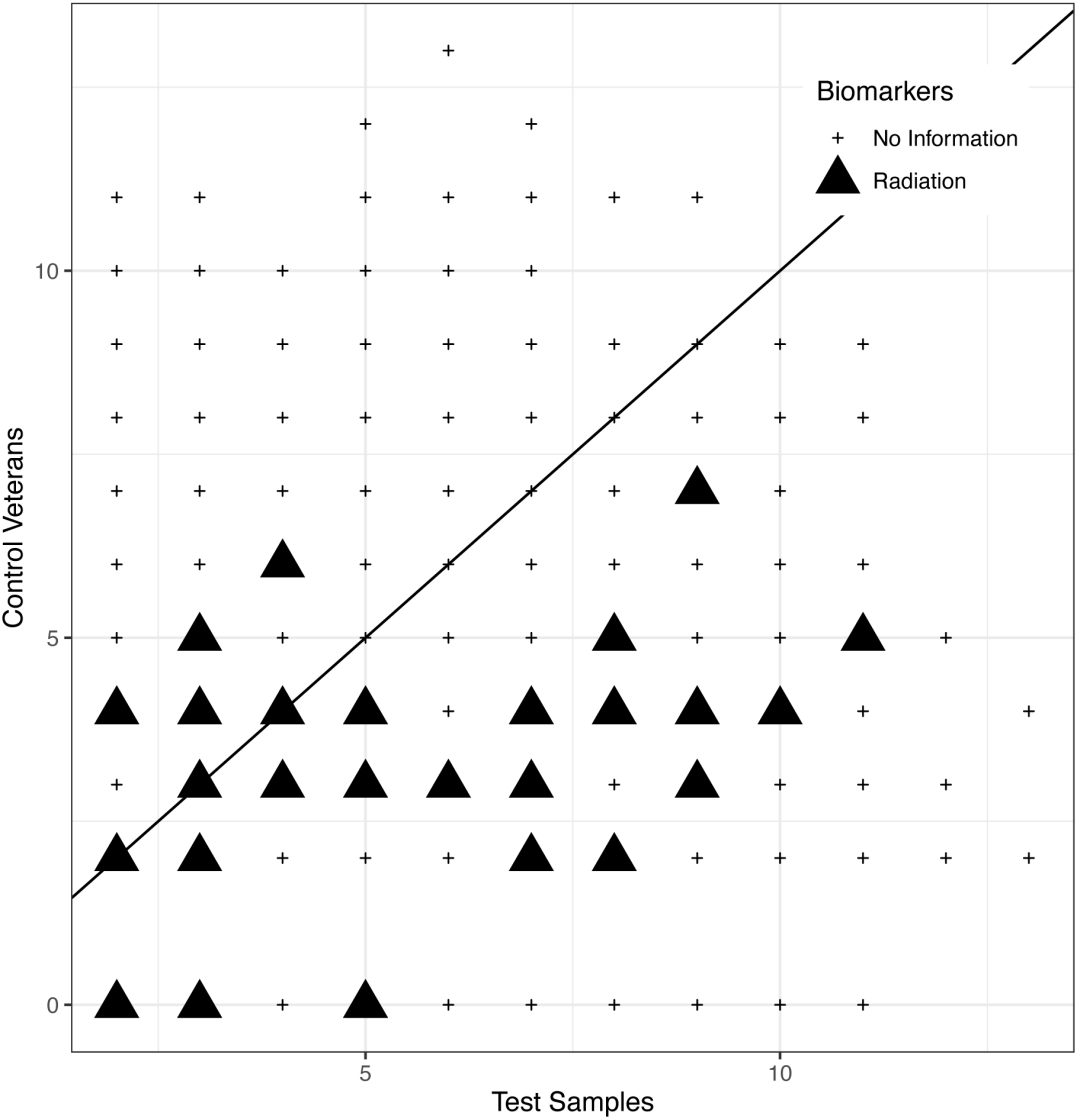
Cohort enrichment of variants in genes associated with exposure to ionising radiation. Distribution of the largest number of samples sharing variants between NT and control veteran cohorts. Each point indicates one of the approximately 60,000 human genes that has a variant in x-axis number of NT samples and y-axis number of control samples. Multiple genes overlap on the same position sharing the same number of mutated NT or control samples. Triangular shaped data points are indicative of genes known to be associated with or known to be markers of ionising radiation exposure.

Several genes have been identified as markers of radiation exposure based on the changes in the transcriptional profiles [38,39]. However, little information is available regarding the molecular underpinnings of the observed expression changes. To explore whether a higher variant frequency is seen in radiation-associated genes in NT veterans compared to controls, we identified a non-exhaustive set of 37 genes from the literature which are annotated as markers of exposure or response to ionising radiation. A summary of these genes is shown Table S4 in S1 File. Using this information we evaluated for any mutational enrichment between the NT and control veteran cohorts using the F2 dataset. This dataset enabled the sensitive evaluation across both cohorts without introducing a potential selection bias by using only cohort specific variants. For this, we counted the total number of veteran samples in each cohort that exhibit at least one variant per gene and compared the mutational frequency in radiation associated genes to that of general gene pool. The results are summarised in Fig 6.

We found that 30 genes associated with ionising radiation showed an increase in mutations in NT veterans, showing variants in one to six more NT samples compared to control. By contrast only 4 radiation-related genes were shown to be more prevalently mutated in the control cohort, presenting INDELs and SNVs in one or two more control samples compared to NT. Further analysis is necessary to understand the relationship between the observed mutational changes and the functional annotation of these genes in relation to radiation exposure.

#### Functional impact of prevalent mutations.

Next, the variants impact on genome function and activity was assessed by looking at the distribution of mutations in annotated regions. To capture the cohort specific effects, we restricted our analysis to variants present only in either NT or control veteran samples (filtered set F3). Using information from GENCODE v47 we annotated each SNV and INDEL present within a gene construct. As such we found 58,535 genes mutated in NT veteran samples compared with 55,379 in control samples. Of these 10,457 displayed variants only in the NT veterans which is a larger group compared to 7,301 genes mutated in the control cohort only.

We further refined our analysis by focusing on variants located in exonic loci only with the aim to identify mutations that could have a direct functional impact altering the gene structure and activity. Overall, we found 6,011 genes mutated in at least 2 NT cohort veterans and in maximum of 1 control group sample. Complementary, we found 6,273 genes mutated in at least 2 control veteran samples and a maximum of 1 NT cohort sample. Of these 3,389 and respectively 3,509 are novel annotations, most likely ncRNAs with no prior functional characterization [40]. A summary of the top gene ontology (GO) terms associated with each group is shown in Tables S12 and 13 in S1 File.

Using the sample specific sets (F3) we were able to obtain a cohort specific picture of the genome variation. However, the filtering process resulted also in a reduction of sensitivity of our data. To try to recover some of this lost sensitivity we constructed an additional sample specific dataset where we kept the variants commonly present in the 1 KG British European individuals. Overall, we identified 3,949,201 and 4,127,471 unique variants present only in NT and control samples respectively. Of these less than 0.008% (NT) and 0.004% (control) are shared between more than a third of veterans (10 samples) in each cohort. Using this data, we once again looked at genes enriched in mutations in the two veteran cohorts.

In the control cohort, two variants (LINC02098-ETS1 and RCL1) were found to be prevalently shared and represented in 14 out of 30 of the veterans. The third most prevalent variant was within the immunoglobulin lambda variable 1–47 (IGLV1–47) gene which shared 13 variants amongst 11 control veteran samples (Table S14 in S1 File).

In the NT veteran cohort, the top two genes, LINC00491 and AC068299.2, each share the same variant among 13 veteran samples (Table S15 in S1 File). The next most prevalent mutated gene was CHODL which presented as a large group of 26 different variants that were shared amongst 12 of the 30 NT veterans sampled. Lastly, the BAZ1A gene was seen to share 45 mutations amongst 11 NT veteran samples.

## Discussion

This study investigated the somatic mutational landscape in an aged population of military veterans using publicly available WGS data. Specifically, this study aimed to identify genetic variations; SNVs, INDELs, in military veterans who had been present at historical nuclear test sites and compare the genetic variation with veterans who had not [10]. Such analysis has the potential to reveal cohort-specific differences, if present, in either the number or type of variant, including those thought to be associated with radiation exposure e.g. INDELs, clustered mutations and/or, differences in the pattern of mutations (SBS signatures) which again may reveal insight into the potential origin of these somatic mutations [2,7].

The number of SNVs and INDELs within both cohorts were seen to align with the expected range for European and British populations [29]. Further, when the control and NT veteran cohorts were compared, we found no elevated occurrences of any variant type, including those reported as radiation signatures, e.g., clustered, INDELs [2]. Although the sample size is small meaning subtle differences in mutation burden may not be detectable, as an aged British population, this lack of genetic variation suggests that both cohorts are strongly influenced by a relatively homogeneous genetic background, in ancestral population and in age. This also implies the veterans were exposed to similar environmental, occupational and/or lifestyle conditions. These findings are consistent with the cytogenetic examination performed as part of the GCFT study which compared NT and control veterans (including the NT and control veterans studied here) whereby, as a cohort, no chromosomal evidence of historical radiation exposure was found in the NT veterans [10,15].

Lawrence et al did however identify a small group of NT veterans with cytogenetic markers consistent with internalised contamination from nuclear fallout (complex chromosome aberrations) and additionally, elevated average frequencies of complex aberrations were weakly associated with an enrichment of germline SNVs allocated to mutation signature SBS16 (SNVs fitted to COSMIC v3.2) [15]. SBS16 is thought to arise via transcription-coupled nucleotide excision repair of bulky DNA lesions [7,8] and although the aetiology remains unknown it is seen in alcohol-associated liver cancers [41]. To explore the possibility of SBS16 reflecting mutational processing from paternal radiation exposure, veterans SNVs were fitted to both COSMIC v3.2 (data not shown) and v3.4. Although SBS16 was seen to be raised in both analyses, we saw no difference between the two cohorts, further, we find SBS1 and SBS5 to be the leading mutational signatures across both cohorts. These signatures are known to correlate with the age of individuals further emphasising the underlying process that dominates the genetic variation in both cohorts as ageing. Interestingly, SBS16 is thought to contaminate SBS5, thus, some SBS16 could be represented in the extremely high count of SBS5. Additionally, given the 1000-fold higher number of unique somatic mutations in the veterans compared to when the same analysis was carried out on the de novo germline mutations [14] it cannot be ruled out that any potential signal present in the veteran samples may have been masked by a large noise level.

Exon variant analysis, focusing on protein-coding genes, lncRNAs, and pseudogenes is important for understanding the functional impact of genetic variants as these regions are usually under stronger selection pressures [42,43]. Our results reflect the previously described conservation with no statistically significant changes between the control and NT samples in the distribution of SNVs and INDELs in annotated exonic regions. Similarly, we observed no significant differences between the control and NT cohort when comparing their cohort-specific mutations (F3 dataset) [44]. We did however see an enrichment of mutations to occur in genes which are associated with ionising radiation exposure and/or response, within the NT veteran cohort. This is an intriguing finding as most of the genes highlighted are described as radiation markers based on their observed changes at transcription level. Whether variants within these genes could also have applications as markers of radiation exposure needs further investigation and validation including with a broader set of radiation associated genes.

The most shared variants within each cohort also enabled qualitative assessment of the overall characteristics of the cohort. For control veterans, one variant in LINC02098-ETS1 was prevalently represented in 14 out of 30 veterans. Although little is currently known about LINC02098, ETS1 is a well-characterised oncogene that functions as a transcription factor involved in various cellular processes, including immune regulation, alopecia (hair loss) and arthritis [45]. Hair loss and arthritis are common features of ageing, thus the prevalence of this mutant in the control cohort likely reflects the advanced age of the veterans. As the NT veterans and controls were age-matched, mutations in this gene were also expected in the NT cohort. Indeed, while the prevalence of a single LINC02098-ETS1 common variant across multiple NT samples was not as prominent, numerous NT mutations at this locus were seen. The second top variant in the control cohort is linked with the RCL1 gene. RCL1 variants could imply an effect on normal ribosomal function and cellular protein production in the aged control cohort. Interestingly, RCL1 has links with neuropsychiatric disorders, problems frequently seen within ageing populations [46,47]. Another gene showing many variants shared across control veteran samples was IGLV1–47. This gene is associated with the immune response where again there is an expectation for mutational enrichment impacting gene function and activity and consequently, the immune response in aging populations [48].

Little is known about the two top variants in the NT veteran cohort. LINC00491 is a long non-coding intergenic RNA [49,50] while AC068299.2 is a novel uncharacterised human gene. The next most prevalent mutated gene, CHODL, has isoforms with a rough average allele frequency of 3.83% in the general British population, based on gnomAD and 1kGP data. This suggests any given CHODL isoform would be expected to appear in around 1 out of every 30 individuals. In the NT veterans, 26 variants associated with different isoforms of CHODL gene were observed in 12 of the 30 veterans which corresponds to ~40% of the cohort. Further, the same variant/s was shared by multiple NT veterans while these variants were not seen within the control cohort (Table S14 and S15 in S1 File). This number of variants in CHODL in a portion of the NT veterans may indicate shared genetic susceptibility possibly because of age, a potential prior environmental exposure, or a role for CHODL in a biological pathway common within these veterans. The CHODL gene encodes chondrolectin, a cell-adhesion protein [51]. Cell adhesion is essential for maintaining tissue structure, mediating cell signalling, and controlling cell migration [52]. Further, CHODL has established functions for maintaining normal nervous system function [51,53]; thus, mutations in CHODL could infer some impact in the normal functioning of motor neurons in muscle control and movement.

The other gene showing a non-statistical enrichment of shared variants in NT veterans was BAZ1A, which encodes the chromatin remodelling factor ACF1. BAZ1A is thought to have a role in various pathways including Wnt signalling as well as in the regulation of genes involved in the development and function of the nervous system [54]. Furthermore, the downregulation of BAZ1A gene has been shown to be associated with cellular senescence [55]. Overall, therefore our findings suggest the advanced cohort age as a key contributor of the observed variant distribution, however other factors, including non-cancer health conditions (the specifics of which are unknown), cannot be excluded.

In conclusion, we found no statistically significant differences in the somatic mutation landscape in a cohort comparison of 30 aged control and 30 NT veterans, based on the filtering strategies applied. SNVs, INDELs and clustering of mutations were consistently distributed across the cohorts and aligned with that seen in larger population studies. Mutational signature analysis showed dominant signatures known to be associated with advanced age. Further, genes identified as being the most prevalently mutated, also suggest ageing to be one of the main drivers in the observed somatic variation. These findings should add further reassurance within this NT community that we see no genetic signatures consistent with radiation exposure nor any difference in overall mutational burden for the veterans sampled.

## Supporting information

S1 File Table S1. Characteristics of the Control and NTV cohorts reproduced from Moorhouse et al 2022. **Table S2**. Summary of the software tools and packages used in variant calling pipeline. **Table S3**. Variant calling thresholds. **Table S4**. Summary of radiation marker genes. The cohort column indicates the enrichment in the NT, control or both cohorts. **Table S5**. All variants identified in the F1, F1 and F3 filtered datasets. **Table S6**. Annotated SNVs in the F1, F1 and F3 filtered datasets. **Table S7**. Annotated INDELs in the F1, F1 and F3 filtered datasets. **Table S8**. Control SBS bootstrap p-value **Table S9**. Control SBS bootstrap errors. **Table S10**. NTV SBS bootstrap p-value. **Table S11**. NTV SBS bootstrap errors. **Table A12**. Gene ontology terms for Control cohort. **Table 13**. Gene ontology terms for nuclear test cohort. **Table 14**. Function impact: Control cohort. **Table 15**. Functional impact: nuclear test cohort. **Figure S1**. Bootstrap signature instability for control samples. **Figure S2**. Bootstrap signature instability for NT samples. **Raw data Figure 1**. **Raw data Figure 2**. **Raw data Figure 3**. **Raw data Figure 4**. **Raw data Figure S1**. **Raw data Figure S2**.
(ZIP)
