# Supplementary material for: Somatic mutation profiles in aged military nuclear test veterans: A comparative whole-genome sequence study
Source: PLoS One. 2026 Jun 30;21(6):e0351624. doi: 10.1371/journal.pone.0351624 (PMC13318011; doi:10.1371/journal.pone.0351624)
Supplement: S1 File — Table S2. Summary of the software tools and packages used in variant calling pipeline. Table S3. Variant calling thresholds. Table S4. Summary of radiation marker genes. The cohort column indicates the enrichment in the NT, control or both cohorts. Table S5. All variants identified in the F1, F1 and F3 filtered datasets. Table S6. Annotated SNVs in the F1, F1 and F3 filtered datasets. Table S7. Annotated INDELs in the F1, F1 and F3 filtered datasets. Table S8. Control SBS bootstrap p-value Table S9. Control SBS bootstrap errors. Table S10. NTV SBS bootstrap p-value. Table S11. NTV SBS bootstrap errors. Table A12. Gene ontology terms for Control cohort. Table 13. Gene ontology terms for nuclear test cohort. Table 14. Function impact: Control cohort. Table 15. Functional impact: nuclear test cohort. Figure S1. Bootstrap signature instability for control samples. Figure S2. Bootstrap signature instability for NT samples. Raw data Figure 1. Raw data Figure 2. Raw data Figure 3. Raw data Figure 4. Raw data Figure S1. Raw data Figure S2. (ZIP) [file pone.0351624.s001.zip › Table S1.docx]

Table S1: Characteristics of the control and NT cohorts reproduced from [1] .

| Variable | Control (N=30) | NTV (N=30) | Statistics |
| --- | --- | --- | --- |
| Age at sampling  (mean (range) years) | 80.0 (76-83) | 79.5 (74-82) | *t* = 1.05; *P* = 0.298 |
| Smoking |  |  |  |
| no | 8 | 8 |  |
| yes | 22 | 22 | - |
| Alcohol consumption | |  |  |
| no | 15 | 13 |  |
| yes | 15 | 17 | *χ*^2^ = 0.27; *P* = 0.605 |
| Occupational radiation exposure | | |  |
| no | 19 | 24 |  |
| yes | 11 | 6 | *χ*^2^ = 2.05; *P* = 0.152 |
| Occupational chemical exposure | | |  |
| no | 7 | 14 |  |
| yes | 23 | 16 | *χ*^2^ = 3.59; *P* = 0.058 |
| Medical diagnostics: number of X-ray | | |  |
| 0 | 0 | 1 |  |
| 1-4 | 11 | 13 |  |
| 5-9 | 8 | 7 |  |
| >10 | 11 | 9 | *χ*^2^ = 1.43; *P* = 0.698 |
| Medical diagnostics: CT-scan | |  |  |
| no | 26 | 26 |  |
| yes | 4 | 4 | - |
| Medical diagnostic: other scans | | |  |
| no | 16 | 13 |  |
| yes | 14 | 17 | *χ*^2^ = 0.60; *P* = 0.438 |
|  |  |  |  |

1. Moorhouse AJ, Scholze M, Sylvius N, Gillham C, Rake C, Peto J, et al. No evidence of increased mutations in the germline of a group of British nuclear test veterans. Sci Rep. 2022 Jul;12[1]:10830. doi:10.1038/s41598-022-14999-w PubMed PMID: 35790751.
