# Supplementary material for: Somatic mutation profiles in aged military nuclear test veterans: A comparative whole-genome sequence study
Source: PLoS One. 2026 Jun 30;21(6):e0351624. doi: 10.1371/journal.pone.0351624 (PMC13318011; doi:10.1371/journal.pone.0351624)
Supplement: S1 File — Table S2. Summary of the software tools and packages used in variant calling pipeline. Table S3. Variant calling thresholds. Table S4. Summary of radiation marker genes. The cohort column indicates the enrichment in the NT, control or both cohorts. Table S5. All variants identified in the F1, F1 and F3 filtered datasets. Table S6. Annotated SNVs in the F1, F1 and F3 filtered datasets. Table S7. Annotated INDELs in the F1, F1 and F3 filtered datasets. Table S8. Control SBS bootstrap p-value Table S9. Control SBS bootstrap errors. Table S10. NTV SBS bootstrap p-value. Table S11. NTV SBS bootstrap errors. Table A12. Gene ontology terms for Control cohort. Table 13. Gene ontology terms for nuclear test cohort. Table 14. Function impact: Control cohort. Table 15. Functional impact: nuclear test cohort. Figure S1. Bootstrap signature instability for control samples. Figure S2. Bootstrap signature instability for NT samples. Raw data Figure 1. Raw data Figure 2. Raw data Figure 3. Raw data Figure 4. Raw data Figure S1. Raw data Figure S2. (ZIP) [file pone.0351624.s001.zip › Table S4.docx]

Table S4. Summary of radiation marker genes. The cohort column indicates the enrichment in the NT, control or both cohorts.

| Gene ID | | Gene Name | | Function | | Cohort | | Reference | |
| --- | --- | --- | --- | --- | --- | --- | --- | --- | --- |
| ANXA2 | Annexin A2 | | Regulation of cellular growth; signal transduction pathways | | NT | | [1] | |  |
| ATF3 | Activating Transcription  Factor 3 | | Response to cellular stress | | NT | | [2] | |  |
| BAX | BCL2 Associated X, Apoptosis Regulator | | Apoptotic activator | | NT | | [3] | |  |
| BBC3 | BCL2 Binding  Component 3 | | Inducer of mitochondrial outer membrane permeabilization and apoptosis | | NT | | [4] | |  |
| BNIP3L | BCL2 Interacting Protein 3 Like | | Inducer of apoptotic changes by targeting mitochondria | | NT | | [5] | |  |
| CDC16 | Cell Division Cycle 16 | | Component of the APC complex; targets cell cycle proteins for degradation | | NT | | [6] | |  |
| CDKN1A | Cyclin Dependent Kinase Inhibitor 1A | | Regulator of cell cycle progression at G1 | | BOTH | | [4] | |  |
| DDB2 | Damage Specific DNA Binding Protein 2 | | DNA nucleotide excision repair | | NT | | [3] | |  |
| DYRK1A | Dual Specificity Tyrosine Phosphorylation Regulated Kinase 1A | | Regulation of cell proliferation and brain development | | NT | | [7] | |  |
| ERCC1 | Excision Repair Cross-Complementing Rodent Repair Deficiency, Complementation Group 1 | | DNA repair | | NT | | [8] | |  |
| ERCC4 | Excision Repair Cross-Complementing Rodent Repair Deficiency, Complementation Group 4 | | DNA repair through a complex with ERCC1 | | NT | | [9] | |  |
| EXO1 | Exonuclease 1 | | Mismatch repair and recombination | | NT | | [10] | |  |
| FAS | Fas Cell Surface Death Receptor | | Apoptotic regulation | | NT | | [11] | |  |
| FDXR | Ferredoxin Reductase | | Initiation of electron transport for cytochromes P450 | | CONTROL | | [3] | |  |
| FHIT | Fragile Histidine Triad Diadenosine Triphosphatase | | Tumour suppressor | | NT | | [12] | |  |
| GADD45A | Growth Arrest And DNA Damage Inducible Alpha | | Response to environmental stress | | NT | | [13] | |  |
| GDF15 | Growth Differentiation Factor 15 | | Gene expression regulation | | NT | | [14] | |  |
| GPX1 | Glutathione Peroxidase 1 | | Protection against oxidative damage | | NT | | [15] | |  |
| HNRNPM | Heterogeneous Nuclear Ribo-nucleoprotein M | | RNA binding protein | | NT | | [16] | |  |
| IL1B | Interleukin 1 Beta | | Mediator of the inflammatory response | | CONTROL | | [17] | |  |
| IL6 | Interleukin 6 | | Inflammation and the maturation of B cells | | BOTH | | [18] | |  |
| ISG15 | ISG15 Ubiquitin Like Modifier | | Chemotactic activity towards neutrophils, cell-to-cell signalling | | NT | | [19] | |  |
| MDM2 | MDM2 Proto-Oncogene | | Promotor of tumour formation | | NT | | [18] | |  |
| PCNA | Proliferating Cell Nuclear Antigen | | DNA replication; DNA repair | | NT | | [20] | |  |
| PHPT1 | Phosphohistidine Phosphatase 1 | | Catalysis of reversible dephosphorylation of histidine residues in proteins | | BOTH | | [21] | |  |
| PRDX1 | Peroxiredoxin 1 | | Antioxidant protective role | | NT | | [22] | |  |
| PRKDC | Protein Kinase, DNA-Activated, Catalytic Subunit | | DNA double strand break repair and recombination | | CONTROL | | [23] | |  |
| RPS27L | Ribosomal Protein S27 Like | | Component of the 40S ribosomal subunit | | NT | | [24] | |  |
| RXRA | Retinoid X Receptor Alpha | | Transcription factor | | NT | | [25] | |  |
| SENS1 | Sestrin 1 | | Cellular response to DNA damage and oxidative stress | | CONTROL | | [26] | |  |
| SOD2 | Superoxide Dismutase 2 | | Protection against superoxide anion radicals | | NT | | [27] | |  |
| STAT3 | Signal Transducer And Activator Of Transcription 3 | | Transcription activator | | NT | | [28] | |  |
| TNF | Tumour Necrosis Factor | | Regulation of cell proliferation, differentiation, apoptosis, lipid metabolism, and coagulation | | NT | | [29] | |  |
| TNFRSF10B | TNF Receptor Superfamily Member 10b | | Transducer of apoptotic signals | | NT | | [30] | |  |
| TNFRSF1A | TNF Receptor Superfamily Member 1A | | Cell survival, apoptosis, inflammation | | NT | | [29] | |  |
| TNFSF10 | TNF Superfamily Member 10 | | Tumour cell apoptosis inducer | | NT | | [31] | |  |

**References:**

1. Waters KM, Stenoien DL, Sowa MB, von Neubeck C, Chrisler WB, Tan R, et al. Annexin A2 modulates radiation-sensitive transcriptional programming and cell fate. Radiat Res. 2013 Jan;179[1]:53–61. doi:10.1667/RR3056.1 PubMed PMID: 23148505.

2. Kool J, Hamdi M, Cornelissen-Steijger P, van der Eb AJ, Terleth C, van Dam H. Induction of ATF3 by ionizing radiation is mediated via a signaling pathway that includes ATM, Nibrin1, stress-induced MAPkinases and ATF-2. Oncogene. 2003 Jul;22[27]:4235–42. doi:10.1038/sj.onc.1206611 PubMed PMID: 12833146.

3. Lee Y, Pujol Canadell M, Shuryak I, Perrier JR, Taveras M, Patel P, et al. Candidate protein markers for radiation biodosimetry in the hematopoietically humanized mouse model. Sci Rep. 2018 Sep;8[1]:13557. doi:10.1038/s41598-018-31740-8 PubMed PMID: 30202043.

4. Badie C, Dziwura S, Raffy C, Tsigani T, Alsbeih G, Moody J, et al. Aberrant CDKN1A transcriptional response associates with abnormal sensitivity to radiation treatment. Br J Cancer. 2008 Jun;98[11]:1845–51. doi:10.1038/sj.bjc.6604381 PubMed PMID: 18493234.

5. Ren Y, Yang P, Li C, Wang WA, Zhang T, Li J, et al. Ionizing radiation triggers mitophagy to enhance DNA damage in cancer cells. Cell Death Discov. 2023 Jul;9[1]:267. doi:10.1038/s41420-023-01573-0 PubMed PMID: 37507394.

6. Zhou PK, Rigaud O. Down-regulation of the human CDC16 gene after exposure to ionizing radiation: a possible role in the radioadaptive response. Radiat Res. 2001 Jan;155[1 Pt 1]:43–9. doi:10.1667/0033-7587[2001]155[0043:drothc]2.0.co;2 PubMed PMID: 11121214.

7. Guard SE, Poss ZC, Ebmeier CC, Pagratis M, Simpson H, Taatjes DJ, et al. The nuclear interactome of DYRK1A reveals a functional role in DNA damage repair. Sci Rep. 2019 Apr;9[1]:6539. doi:10.1038/s41598-019-42990-5 PubMed PMID: 31024071.

8. Huang YJ, Huang MY, Cheng TL, Kuo SH, Ke CC, Chen YT, et al. ERCC1 Overexpression Increases Radioresistance in Colorectal Cancer Cells. Cancers Basel. 2022 Sep;14[19]:4798. doi:10.3390/cancers14194798 PubMed PMID: 36230725.

9. Shi R, Wang S, Jiang Y, Zhong G, Li M, Sun Y. ERCC4: a potential regulatory factor in inflammatory bowel disease and inflammation-associated colorectal cancer. Front Endocrinol Lausanne. 2024;15:1348216. doi:10.3389/fendo.2024.1348216 PubMed PMID: 38516408.

10. He D, Li T, Sheng M, Yang B. Exonuclease 1 [Exo1] Participates in Mammalian Non-Homologous End Joining and Contributes to Drug Resistance in Ovarian Cancer. Med Sci Monit. 2020 Mar;26:e918751. doi:10.12659/MSM.918751 PubMed PMID: 32167078.

11. Albanese J, Dainiak N. Ionizing radiation alters Fas antigen ligand at the cell surface and on exfoliated plasma membrane-derived vesicles: implications for apoptosis and intercellular signaling. Radiat Res. 2000 Jan;153[1]:49–61. doi:10.1667/0033-7587[2000]153[0049:irafal]2.0.co;2 PubMed PMID: 10630977.

12. Lu L, Hu B, Yu F, Wang Y. Low dose radiation-induced adaptive response preventing HPRT mutation is Fhit independent. Int J Radiat Biol. 2009 Jun;85[6]:532–7. doi:10.1080/09553000902883828 PubMed PMID: 19401904.

13. Broustas CG, Mukherjee S, Shuryak I, Taraboletti A, Angdisen J, Ake P, et al. Impact of GADD45A on Radiation Biodosimetry Using Mouse Peripheral Blood. Radiat Res. 2023 Sep;200[3]:296–306. doi:10.1667/RADE-23-00052.1 PubMed PMID: 37421415.

14. Sándor N, Schilling-Tóth B, Kis E, Benedek A, Lumniczky K, Sáfrány G, et al. Growth Differentiation Factor-15 [GDF-15] is a potential marker of radiation response and radiation sensitivity. Mutat Res Genet Toxicol Env Mutagen. 2015 Nov;793:142–9. doi:10.1016/j.mrgentox.2015.06.009 PubMed PMID: 26520384.

15. Zhang S, Zhang G, Wang P, Wang L, Fang B, Huang J. Effect of Selenium and Selenoproteins on Radiation Resistance. Nutrients. 2024 Aug;16[17]:2902. doi:10.3390/nu16172902 PubMed PMID: 39275218.

16. Haley B, Paunesku T, Protić M, Woloschak GE. Response of heterogeneous ribonuclear proteins [hnRNP] to ionising radiation and their involvement in DNA damage repair. Int J Radiat Biol. 2009 Aug;85[8]:643–55. doi:10.1080/09553000903009548 PubMed PMID: 19579069.

17. Subedi P, Gomolka M, Moertl S, Dietz A. Ionizing Radiation Protein Biomarkers in Normal Tissue and Their Correlation to Radiosensitivity: A Systematic Review. J Med. 2021 Feb;11[2]:140. doi:10.3390/jpm11020140 PubMed PMID: 33669522.

18. Mzizi Y, Mbambara S, Moetlhoa B, Mahapane J, Mdanda S, Sathekge M, et al. Ionising radiation exposure-induced regulation of selected biomarkers and their impact in cancer and treatment. Front Nucl Med. 2024;4:1469897. doi:10.3389/fnume.2024.1469897 PubMed PMID: 39498386.

19. Wardlaw CP, Petrini JHJ. ISG15: A link between innate immune signaling, DNA replication, and genome stability. Bioessays. 2023 Jul;45[7]:e2300042. doi:10.1002/bies.202300042 PubMed PMID: 37147792.

20. Koturbash I, Merrifield M, Kovalchuk O. Fractionated exposure to low doses of ionizing radiation results in accumulation of DNA damage in mouse spleen tissue and activation of apoptosis in a p53/Atm-independent manner. Int J Radiat Biol. 2017 Feb;93[2]:148–55. doi:10.1080/09553002.2017.1231943 PubMed PMID: 27758128.

21. Tichy A, Kabacik S, O’Brien G, Pejchal J, Sinkorova Z, Kmochova A, et al. The first in vivo multiparametric comparison of different radiation exposure biomarkers in human blood. PLoS One. 2018;13[2]:e0193412. doi:10.1371/journal.pone.0193412 PubMed PMID: 29474504.

22. Jezierska-Drutel A, Attaran S, Hopkins BL, Skoko JJ, Rosenzweig SA, Neumann CA. The peroxidase PRDX1 inhibits the activated phenotype in mammary fibroblasts through regulating c-Jun N-terminal kinases. BMC Cancer. 2019 Aug;19[1]:812. doi:10.1186/s12885-019-6031-4 PubMed PMID: 31419957.

23. Tsuji AB, Sudo H, Sugyo A, Otsuki M, Miyagishi M, Taira K, et al. A fast, simple method for screening radiation susceptibility genes by RNA interference. Biochem Biophys Res Commun. 2005 Aug;333[4]:1370–7. doi:10.1016/j.bbrc.2005.06.047 PubMed PMID: 15979584.

24. Zhao Y, Tan M, Liu X, Xiong X, Sun Y. Inactivation of ribosomal protein S27-like confers radiosensitivity via the Mdm2-p53 and Mdm2-MRN-ATM axes. Cell Death Dis. 2018 Feb;9[2]:145. doi:10.1038/s41419-017-0192-3 PubMed PMID: 29396424.

25. Rajamani BM, Illangeswaran RSS, Benjamin ESB, Balakrishnan B, Jebanesan DZP, Das S, et al. Modulating retinoid-X-receptor alpha [RXRA] expression sensitizes chronic myeloid leukemia cells to imatinib in vitro and reduces disease burden in vivo. Front Pharmacol. 2023;14:1187066. doi:10.3389/fphar.2023.1187066 PubMed PMID: 37324449.

26. Albrecht H, Durbin-Johnson B, Yunis R, Kalanetra KM, Wu S, Chen R, et al. Transcriptional response of ex vivo human skin to ionizing radiation: comparison between low- and high-dose effects. Radiat Res. 2012 Jan;177[1]:69–83. doi:10.1667/rr2524.1 PubMed PMID: 22029842.

27. Zhang Z, Lang J, Cao Z, Li R, Wang X, Wang W. Radiation-induced SOD2 overexpression sensitizes colorectal cancer to radiation while protecting normal tissue. Oncotarget. 2017 Jan;8[5]:7791–800. doi:10.18632/oncotarget.13954 PubMed PMID: 27999194.

28. Wang X, Zhang X, Qiu C, Yang N. STAT3 Contributes to Radioresistance in Cancer. Front Oncol. 2020;10:1120. doi:10.3389/fonc.2020.01120 PubMed PMID: 32733808.

29. Hallahan DE, Spriggs DR, Beckett MA, Kufe DW, Weichselbaum RR. Increased tumor necrosis factor alpha mRNA after cellular exposure to ionizing radiation. Proc Natl Acad Sci U A. 1989 Dec;86[24]:10104–7. doi:10.1073/pnas.86.24.10104 PubMed PMID: 2602359.

30. Ishii K, Ishiai M, Morimoto H, Kanatsu-Shinohara M, Niwa O, Takata M, et al. The Trp53-Trp53inp1-Tnfrsf10b pathway regulates the radiation response of mouse spermatogonial stem cells. Stem Cell Rep. 2014 Oct;3[4]:676–89. doi:10.1016/j.stemcr.2014.08.006 PubMed PMID: 25358794.

31. Ivanov VN, Hei TK. A role for TRAIL/TRAIL-R2 in radiation-induced apoptosis and radiation-induced bystander response of human neural stem cells. Apoptosis. 2014 Mar;19[3]:399–413. doi:10.1007/s10495-013-0925-4 PubMed PMID: 24158598.

32. Zhao J, Zhi Z, Zhang M, Li Q, Li J, Wang X, et al. Predictive value of single nucleotide polymorphisms in XRCC1 for radiation-induced normal tissue toxicity. Onco Targets Ther. 2018;11:3901–18. doi:10.2147/OTT.S156175 PubMed PMID: 30013370.
